# Supplementary figures and images for: Seasonal Variations in Production Performance, Health Status, and Gut Microbiota of Meat Rabbit Reared in Semi-Confined Conditions
Source: Animals (Basel). 2023 Dec 28;14(1):113. doi: 10.3390/ani14010113 (PMC10778228; doi:10.3390/ani14010113)

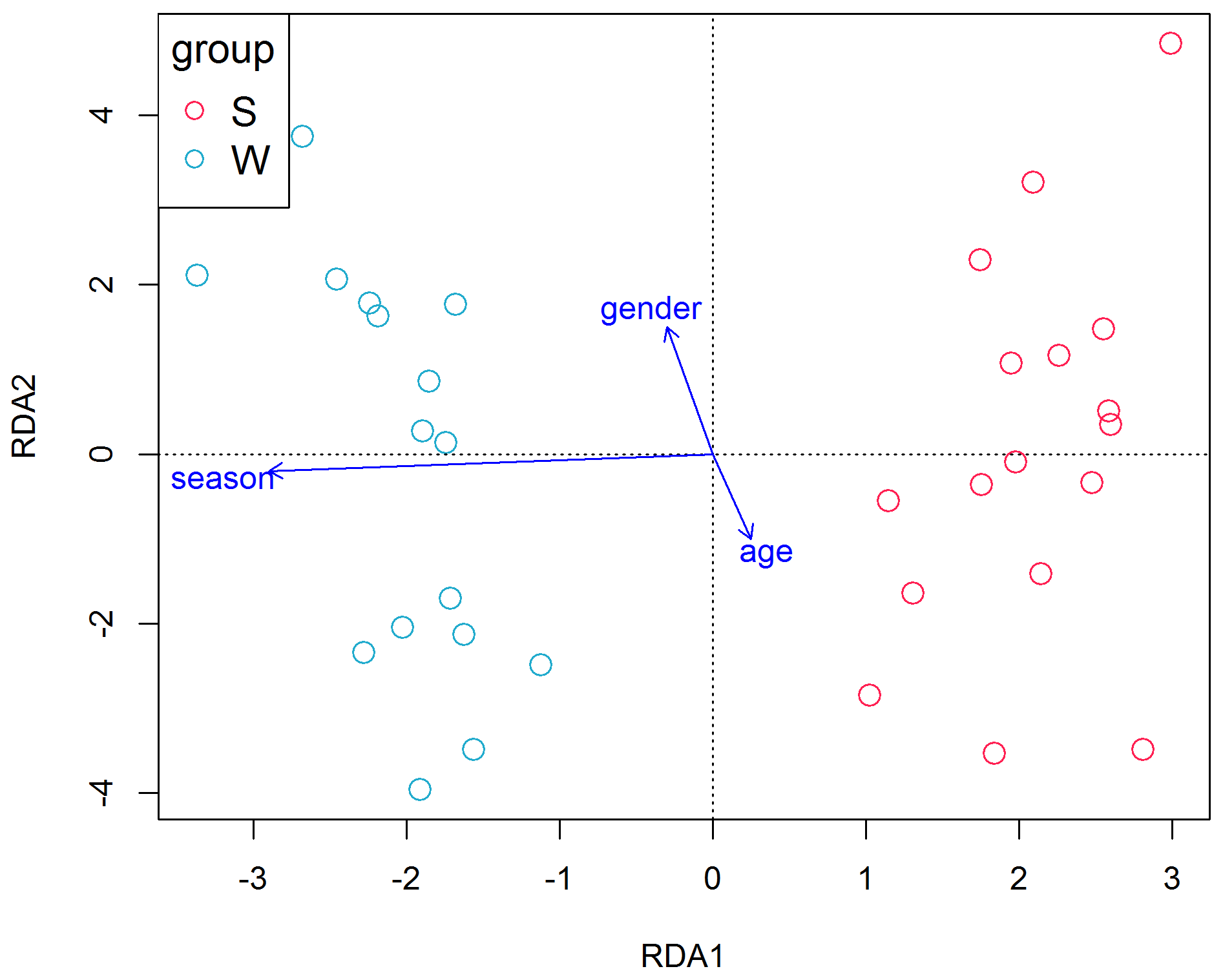

Supplement: Supplementary file 1 [file animals-14-00113-s001.zip › Fig S1.tiff]
